# Glycinergic Inhibition Targets Specific Off Cone Bipolar Cells in Primate Retina

**DOI:** 10.1523/ENEURO.0432-20.2020

**Published:** 2021-02-23

**Authors:** Amanda J. McLaughlin, Kumiko A. Percival, Jacqueline Gayet-Primo, Teresa Puthussery

**Affiliations:** 1 School of Optometry, University of California, Berkeley, Berkeley, CA 94720; 2 Helen Wills Neuroscience Institute, University of California, Berkeley, Berkeley, CA 94720; 3 Vision Science Graduate Program, University of California, Berkeley, Berkeley, CA 94720; 4 Casey Eye Institute, Oregon Health and Science University, Portland, OR 97239

**Keywords:** bipolar cell, glycine receptors, ion channels, macaque, retina, rod pathway

## Abstract

Adapting between scotopic and photopic illumination involves switching the routing of retinal signals between rod and cone-dominated circuits. In the daytime, cone signals pass through parallel On and Off cone bipolar cells (CBCs), that are sensitive to increments and decrements in luminance, respectively. At night, rod signals are routed into these cone-pathways via a key glycinergic interneuron, the AII amacrine cell (AII-AC). AII-ACs also provide On-pathway-driven crossover inhibition to Off-CBCs under photopic conditions. In primates, it is not known whether all Off-bipolar cell types receive functional inputs from AII-ACs. Here, we show that select Off-CBC types receive significantly higher levels of On-pathway-driven glycinergic input than others. The rise and decay kinetics of the glycinergic events are consistent with involvement of the α1 glycine receptor (GlyR) subunit, a result supported by a higher level of GLRA1 transcript in these cells. The Off-bipolar types that receive glycinergic input have sustained physiological properties and include the flat midget bipolar (FMB) cells, which provide excitatory input to the Off-midget ganglion cells (GCs; parvocellular pathway). Our results suggest that only a subset of Off-bipolar cells have the requisite receptors to respond to AII-AC input. Taken together with results in mouse retina, our findings suggest a conserved motif whereby signal output from AII-ACs is preferentially routed into sustained Off-bipolar signaling pathways.

## Significance Statement

Visual signals pass through different retinal neurons depending on the prevailing level of illumination. The AII amacrine cells (AII-ACs) are a key inhibitory neuron involved in signaling during daytime and nighttime vision. Here, we show that only select Off-bipolar cell types are equipped with receptors to receive signals from AII-ACs. These results suggest that rod signals may reach the brain via specific output channels. Our results further our understanding of how visual signals are routed through retinal circuits during nighttime and daytime vision.

## Introduction

**Figure 1.**
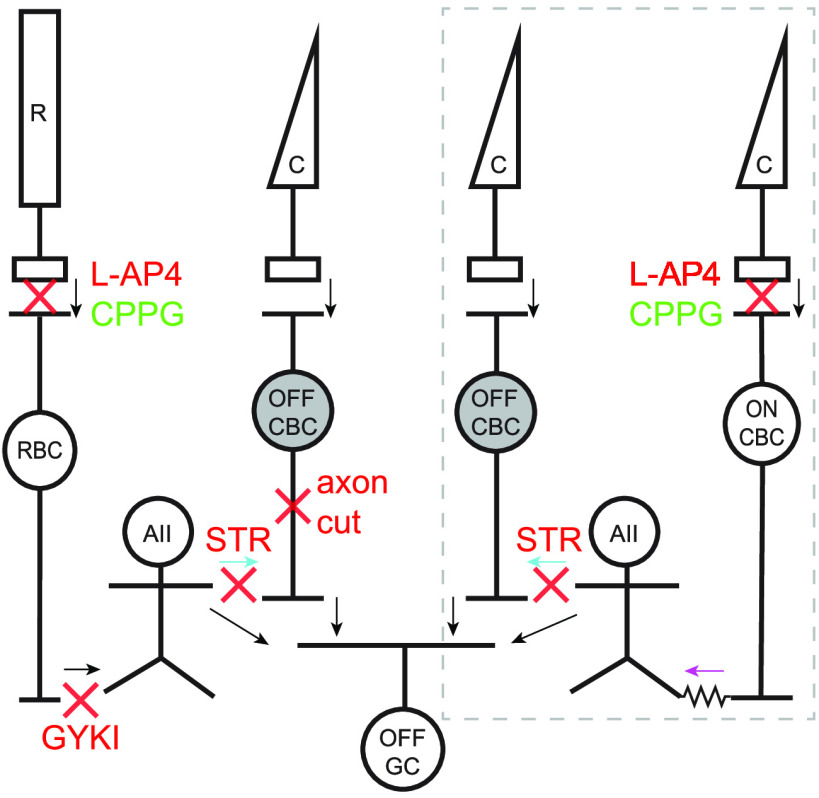
Schematic showing glycinergic inhibition onto Off-CBCs through rod and cone pathways. Arrows indicate direction of signal flow and red crosses indicate site of action of pharmacological agents used herein. Under scotopic conditions, light signals from rods (R) are transmitted to rod bipolar cells (RBCs) via mGluR6 receptors. This synapse can be blocked with L-AP4 and activated with CPPG. RBCs transmit signals to AII-ACs (AII) via GYKI-sensitive AMPA receptors. AII-ACs inhibit Off-CBCs through glycinergic synapses (cyan arrows) that can be blocked with STR. Under photopic conditions (gray dotted box), cones (C) signal light increments to On-CBCs via mGluR6 receptors. On-CBCs drive AII-ACs via gap junctions (magenta arrow) and AII-ACs in turn inhibit Off-CBCs via glycinergic synapses. In both pathways, signals from Off-CBCs are relayed to Off-GCs (OFF GCs). AII-ACs can also signal directly to some OFF GC types.

Understanding human retinal function requires knowledge of the synaptic and circuit mechanisms that are engaged under different illumination levels. Signals from rod photoreceptors are routed through different circuits depending on illumination level (Müller et al., 1988; Grimes et al., 2018b). Under scotopic (nighttime) light levels, the primary rod pathway transmits signals from rods to rod bipolar cells, which in turn signal to AII amacrine cells (AII-ACs). The AII-ACs then relay signals to Off-cone bipolar cells (CBCs) through sign-inverting glycinergic synapses, to On-CBCs through sign-conserving gap-junctions (Kolb and Famiglietti, 1974; Famiglietti and Kolb, 1975; Bloomfield and Dacheux, 2001), or directly to Off-ganglion cells (Off-GCs; Fig. 1; Murphy and Rieke, 2008; Arman and Sampath, 2012; Beaudoin et al., 2019). In this way, increments and decrements in light intensity detected by the rods, and collected by the On-type rod bipolar cells, are routed appropriately into the On and Off pathways. As light levels rise into the mesopic (twilight) range, rod signals pass to cones via gap junctions (secondary rod pathway) and are then transmitted through the CBC pathways (Nelson, 1977; Schneeweis and Schnapf, 1995, 1999). A tertiary rod pathway also operates at mesopic light levels, in which rods synapse directly with Off-CBCs (Soucy et al., 1998; Hack et al., 1999; Tsukamoto et al., 2001; Li et al., 2010).

A recent study showed that primates differ from other species in that rod signals are routed principally through the primary rod pathway, via AII-ACs, even at mesopic light levels (Grimes et al., 2018a). However, it is not known whether all of the five primate Off-CBC types (Boycott and Wässle, 1991; Haverkamp et al., 2003; Puthussery et al., 2013, 2014; Peng et al., 2019) receive AII-AC input and, thus, participate in primary rod pathway signaling. Ultrastructural studies in mice show that the majority of glycinergic output synapses from AII-ACs are directed toward a single Off-CBC subtype (CBC2; Tsukamoto and Omi, 2017; Graydon et al., 2018; Gamlin et al., 2020). If glycinergic inhibition from primate AII-ACs is similarly biased toward specific Off-CBC types, the temporal properties of rod signals would be shaped by these bipolar cells. Indeed, anatomic studies in macaque suggest flat midget bipolar (FMB) cells as a major recipient of AII-AC output synapses (Grünert and Wässle, 1996).

Routing of signals from AII-AC to Off-CBCs also occurs under photopic light levels, when rods are saturated. Under these conditions, cones tonically depolarize On-CBCs, which in turn depolarize the AII-ACs via gap junctions such that they increase their glycinergic output onto Off-CBC axon terminals (Liang and Freed, 2010). Such “crossover” inhibition from the On pathway hyperpolarizes the Off-CBCs below threshold for glutamate release and thus rectifies their output (Liang and Freed, 2010). Understanding On-pathway-driven glycinergic input to Off-CBCs is thus also relevant for signal routing under photopic conditions.

Here, we use electrophysiological recordings to study glycinergic inputs to primate Off-CBCs. We find evidence that FMB and DB1 cells receive higher levels of α1-subunit mediated glycinergic input than other Off-CBC types, a result corroborated by evidence for higher levels of GLRA1 transcript in these cells. Given that glycine receptors (GlyRs) are necessary for AII-AC to Off-CBC signaling, the low levels of GlyR expression in some Off-CBC types, suggests there may be heterogeneity in the scotopic sensitivity and linearity of primate Off-CBCs and, by extension, their postsynaptic GC partners.

## Materials and Methods

### Tissue preparation

Eyes from adult rhesus macaques (*Macaca mulatta*) of either sex were collected from animals euthanized for unrelated projects at the Oregon or California National Primate Research Centers. Eyes were enucleated immediately postmortem, the anterior eye and vitreous were removed, and posterior eyecups were transported in bicarbonate-buffered Ames’ medium (US Biologicals) equilibrated with 95% O_2_/5% CO_2_ at 22–25°C (pH 7.4). After ∼1 h, the retina/RPE/choroid complex was dissected free of the sclera and maintained in the same buffer until slices were made. For slice preparation, the neural retina was isolated from the RPE/choroid and ∼300-μm-thick slices were made using a vibrating blade microtome or tissue chopper. For vibratome sections, retinas were embedded in 3% low-melting point agar in HEPES-Ames’ (pH 7.4). All samples were from peripheral (>8 mm) superonasal or inferonasal retina. Slices were prepared and recordings made under low photopic background illumination levels.

### Immunohistochemistry

Macaque retinae were briefly perfusion-fixed or immersion-fixed in 4% paraformaldehyde in 0.1 m PB or PBS for 30–60 min. After fixation, tissues were rinsed in PBS, cryoprotected in graded sucrose solutions (10%, 20%, 30%) and stored at −20°C until use. Retinal pieces were embedded in Cryo-gel embedding medium and cryosectioned at 14–16 μm. For immunohistochemistry, sections were blocked in 10% normal horse serum, 0.3–1% Triton X-100, 0.025% NaN_3_ in PBS for 1 h. Primary antibodies were diluted in 3% normal horse serum, 0.3–1% Triton X-100, 0.025% NaN_3_ in PBS and applied overnight at 20–25°C. The primary antibodies were: ms anti-Kir2.1 (Clone N112B/14, Neuromab, catalog #73-210, tissue culture supernatant, RRID: AB_11001668, dilution 1:15), ms anti-Ca_V_3.1 (Clone N178A/9; catalog #Neuromab, 73-206, tissue culture supernatant, RRID: AB_10673097, dilution 1:10), sheep anti-secretagogin (BioVendor, RD1884120100, RRID: AB_2034060, dilution 1:2000), and rabbit anti-GLT-1 (Tocris, #2063, dilution 1:2000). Secondary antibodies against mouse, rabbit, or sheep antigens were raised in donkey and conjugated to Alexa Fluor 488 or Alexa Fluor 594 (Invitrogen, ThermoFisher, RRIDs: AB_141607, AB_2534083, AB_141637). Samples were mounted in Mowiol. Confocal images were acquired on an Olympus FV1000 (60/1.4 objective, 488-, 559-, and 643-nm laser lines) or a Zeiss LSM 880 laser scanning microscope (63/1.4 objective, 488-, 561-, and 594-nm laser lines). Images were acquired sequentially to prevent fluorescent cross talk. In some cases, adjustments to image brightness and contrast were made with ImageJ (Fiji).

### Bipolar cell electrophysiology

For whole-cell patch-clamp recordings, retinas were superfused with warm (31–33°C) bicarbonate-buffered Ames’ medium at ∼2–3 ml/min. Patch electrodes (9–12 MΩ) were coated in Parafilm to reduce pipette capacitance. Electrodes were filled with an intracellular solution containing the following: 130 mm K-methanesulfonic acid, 8 mm KCl, 2 mm Mg_2_-ATP, 1 mm Na-GTP, 1 mm EGTA, 10 mm Na_0.5_-HEPES, and ∼0.1 mm Alexa Fluor 488 hydrazide adjusted to pH 7.35 with KOH (osmolarity = 290 mOsm). In some cases, 10 mm phospho-creatine was included in the intracellular solution. To assess recovery from inactivation for T-type calcium currents, a Cs^+^-based intracellular solution was used to block voltage-gated potassium current. This solution contained the following: 120 mm Cs-methanesulfonic acid, 8 mm KCl, 2 mm Mg_2_-ATP, 1 mm Na-GTP, 1 mm EGTA, 10 mm Na_0.5_-HEPES, 10 mm phosophocreatine, and ∼0.1 mm Alexa Fluor 488 hydrazide adjusted to pH 7.35 with CsOH (osmolarity = 290 mOsm). Reported voltages were corrected for a liquid junction potential of −10 mV unless otherwise indicated. Currents were filtered at a −3-dB cutoff frequency of 2 kHz by the four-pole Bessel filter of a HEKA EPC-10 double patch amplifier, and digitized at 10 kHz. Series resistance was compensated online and monitored during recordings. Cells were excluded from analysis if series resistance was >35 MΩ.

For pharmacological experiments, stock solutions were made in ultrapure water and aliquots stored at −20°C until further use. GYKI-53655, L-AP4 and tetrodotoxin were obtained from Tocris Bioscience, and strychnine (STR) was obtained from Sigma. For all pharmacological experiments, drugs were superfused for at least 3 min to ensure that steady-state drug effects had been reached. CPPG (Tocris Bioscience) was diluted in HEPES-buffered Ames’ medium and focally applied to the outer plexiform layer via a ∼5-MΩ patch pipette. For these experiments, bath flow was directed toward the outer retina to obviate drug effects on the inner retinal circuitry.

### Data analysis

#### Current variance

**Figure 2.**
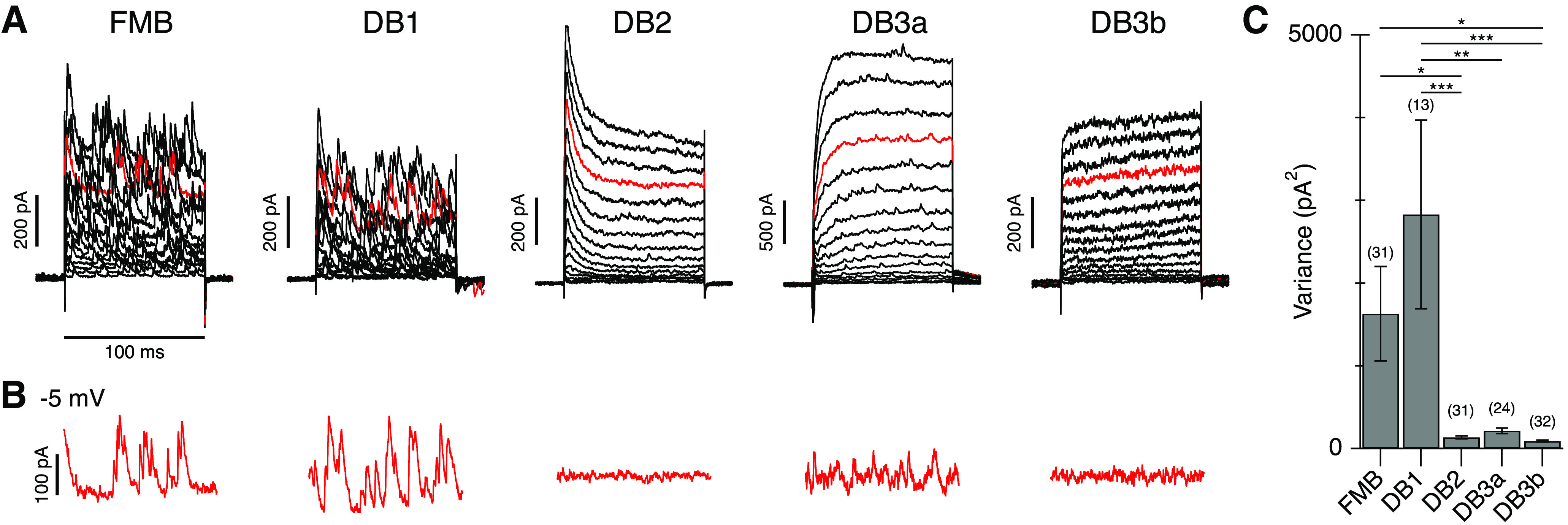
Spontaneous inhibition is present in specific Off-CBC types. ***A***, Spontaneous inhibitory events in representative FMB, DB1, DB2, DB3a, and DB3b cells. The stimulus was a series of depolarizing voltage steps from a holding potential of −70 mV (+5-mV increments, 16 steps). The red traces show currents at – 5 mV near the calculated excitatory reversal potential of −7 mV. ***B***, Trend-subtracted currents during the step to –5 mV from the same cells shown in ***A***. ***C***, Bar graph showing average current variance in the different Off-CBCs during a voltage step to −5 mV. Number of cells for each type is indicated above bars. Data are mean ± SEM; *p < 0.01, **p < 0.01, ***p < 0.001 with Tukey’s *post hoc* tests after one-way ANOVA.

The current variance measurements in Figure 2 were obtained during a voltage-step to −5 mV. To exclude contributions from time-varying voltage-gated current components (e.g., A-type potassium currents), traces were trend-subtracted by fitting a single exponential to the voltage-activated current component (Fig. 2*B*). For variance measurements before and after puff application of CPPG, a 0.5-s time window was analyzed before and during drug application.

#### IPSC analysis and pharmacology

sIPSCs were detected and extracted from recordings using a matched template filter approach implemented in Igor Pro 8. First, average event templates were obtained using an amplitude threshold and average event rise and decay times. A matched template filter was then used to convolve the data traces with the templates; 10–90% rise times were measured from a sigmoidal fit and IPSC decay time constants were determined using a single exponential fit. For quantification of the effects of STR, L-AP4, and GYKI-53655 on sIPSCs, IPSC event frequency was estimated using an amplitude threshold cutoff of 2 SDs above the mean current level. Only events with a minimum width of 1 ms were counted to prevent detection of non-neural noise.

#### Voltage-gated currents

To obtain the net voltage-activated component of the membrane current, scaled linear current components were subtracted from the raw recorded currents. To obtain empirical estimates of the activation range and voltage-dependence of voltage-gated currents, leak-subtracted current–voltage (I-V) relations were fit using a Boltzmann equation, and assuming ohmic conductance:

$$I_{m}=G_{max}\left(V_{m}-E_{ion}\right)/\left(1+e^{\left(\left(V_{0.5}-V_{m}\right)/z\right)}\right),$$

where, *G_max_* is the maximum conductance, *E_ion_* is the reversal potential, *V_0.5_* is the half-activation potential, and *z* is the voltage-sensitivity. All analysis was performed with custom routines in Igor Pro 7 or 8 (Wavemetrics).

The time constant of T-type current activation was measured by fitting a sigmoid to the activation phase and calculating the 10–90% rise time. The inactivation time course was assessed by fitting the decay of the current waveforms at the peak potential of the I-V relationships with a single exponential function. Recovery from inactivation for T-type currents was measured using a paired-pulse protocol. Cells were depolarized with a 500-ms prepulse from −90 to −40 mV, and a second test pulse (−90 to −40 mV, 100 ms) was given at increasing time intervals after the prepulse. Recovery was expressed as the amplitude of the test pulse as a fraction of the prepulse amplitude. The time constant of recovery from inactivation was measured by fitting a double-exponential function where τ_1_ and τ_2_ represent a fast and slow component of recovery.

### Visualizations of single-cell RNA sequencing data

Data mining and visualization of single-cell RNA-sequencing expression profiles from peripheral primate bipolar cells (Peng et al., 2019) was performed using the Broad Institute Single Cell Portal (https://singlecell.broadinstitute.org/single_cell).

### Experimental design and statistical analysis

Statistical comparisons were made using one-way ANOVA, unpaired *t* tests, or the Wilcoxon signed-rank test as indicated. For each experiment, the number of cells is indicated in the text or in the figure legends. Data were tested for normality and Welch’s correction was applied when variances were unequal. An α level of 0.05 was used for statistical significance. All statistical analyses were performed in Igor Pro 8.

## Results

### Prominent spontaneous inhibitory input in a subset of Off-CBC types

Our first objective was to determine whether specific Off-CBC types in primate retinas receive higher levels of glycinergic input than others. There are five Off-CBC types in macaque and human retina, which differ in their morphology, transcriptomic profiles, and functional properties (Boycott and Wässle, 1991; Haverkamp et al., 2003; Puthussery et al., 2013, 2014; Peng et al., 2019). In this study, we distinguished Off-CBC types by their voltage-activated currents (Puthussery et al., 2013; Figs. 2*A*) and by including a fluorescent dye in the recording electrode to visualize morphology and axon terminal stratification at the end of the recordings (Puthussery et al., 2013, 2014). Figure 2*A* shows a comparison of spontaneous currents in the different Off-CBC types during depolarizing steps from a holding potential of −70 mV. DB1 and FMB cells exhibited frequent, large spontaneous postsynaptic currents. Smaller and less frequent events were observed in DB3a cells, while events were rarely observed in DB2 and DB3b cells. The spontaneous currents persisted at the excitatory reversal potential, indicating an inhibitory origin (Fig. 2*A*, red traces, *B*). After subtracting time-dependent voltage activated currents, we found there was a significant difference in current variance between cell types, reflecting in large part the difference in sIPSC frequency (one-way ANOVA, dF = 4.0, *F* = 7.0, *p* = 4.0e-5; Fig. 1*C*). These results suggest that specific Off-CBC types receive more spontaneous inhibition than others. Similar large-amplitude sIPSCs have been observed in Off-CBC cells of other mammalian retinas and have been shown to be of glycinergic origin (Ivanova et al., 2006; Graydon et al., 2018). Our next goal was to test whether the observed sIPSCs were glycinergic and to investigate the circuit origin of these events (Fig. 1, schematic).

### Spontaneous events are glycinergic and arise at the level of the axon terminals

**Figure 3.**
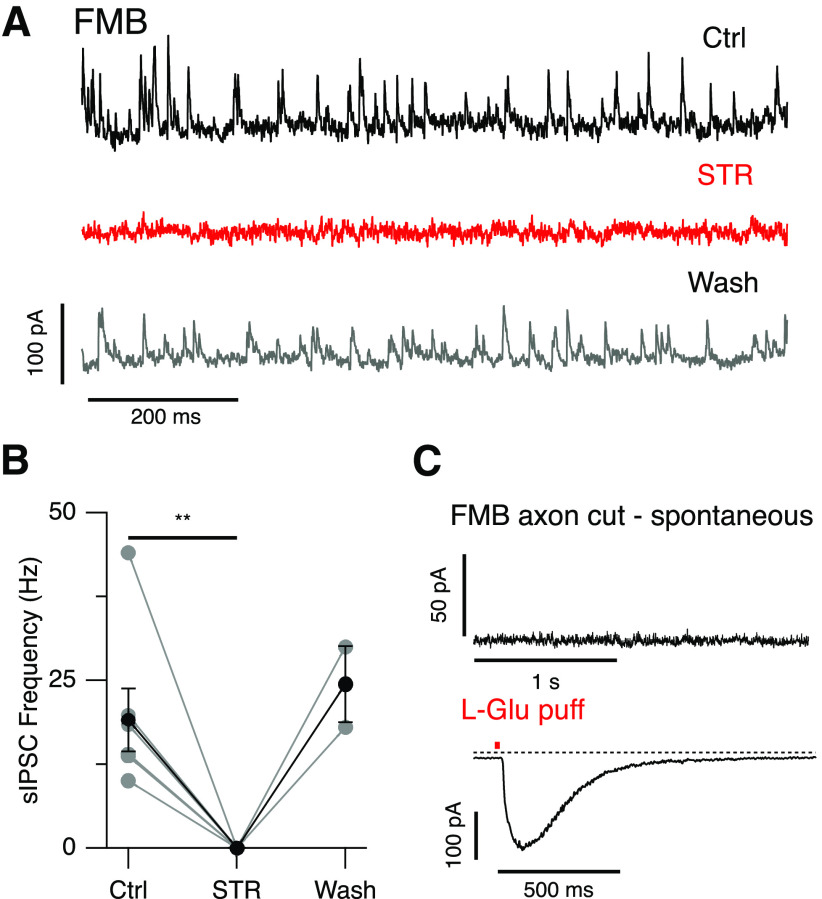
Spontaneous inhibition in FMB cells is glycinergic and arises at the axon terminals. ***A***, Example traces from an FMB cell showing the frequency of sIPSCs before, during, and after bath application of STR (0.5 μm). Events were recorded during a voltage step to −20 mV from a holding potential of −70 mV. ***B***, Effect of STR on sIPSC frequency for a group of FMB cells (Ctrl and STR; *n* = 7 cells, washout; *n* = 2 cells, **p = 0.004, Wilcoxon signed-rank test). Gray points indicate individual cells, black points indicate mean ± SEM. ***C***, top panel, Example trace showing a lack of spontaneous events in an axotomized FMB cell during a voltage step to −20 mV from −70 mV. Bottom panel, Current response to a 20-ms puff application (red bar) of L-glutamate (1 mm) applied to the same cell. The presence of a glutamate-evoked inward current confirms the Off-type physiology of the axotomized midget cell. Holding potential is −70 mV.

Application of the GlyR antagonist, STR (0.5 μm) completely abolished the large sIPSCs in FMB cells (Ctrl 19.1 ± 11.5 vs STR 0.0 ± 0.0 Hz, *n* = 7 cells, mean ± SD, *p* = 0.004, Wilcoxon signed-rank test; Fig. 3*A*,*B*). The effect of STR was slowly reversible on washout, and partial or complete recovery was achieved in two cells (Fig. 3*B*). If the sIPSCs arise from glycinergic synapses on the axon terminals of Off-CBCs, then they should be absent in axotomized cells. To test this prediction, we recorded from midget bipolar cells in which axons had been cut during slice preparation (Fig. 3*C*). In the absence of an axon terminal, midget bipolar cells could be identified by their distinctive dendritic morphology because they contact a single cone pedicle. We also distinguished axonless On-midget bipolar (IMB) from Off-midget bipolar (FMB) cells by their responses to L-glutamate puffed onto their dendrites. FMB cells exhibited an inward current to L-glutamate application (Fig. 3*C*), consistent with activation of ionotropic glutamate receptors (Puthussery et al., 2014). None of the five axotomized FMB cells exhibited sIPSCs, suggesting that glycinergic inputs arise at the level of the axon terminals. Similar experiments were not performed in DB1 cells as these could not be unequivocally distinguished in their axotomized form and their sparsity precluded pharmacological experiments. Thus, we next tested for similarities in activation and kinetic properties of the observed sIPSCs in FMB and DB1 cells.

### Spontaneous IPSCs have kinetics consistent with α1 subunit containing GlyRs

**Figure 4.**
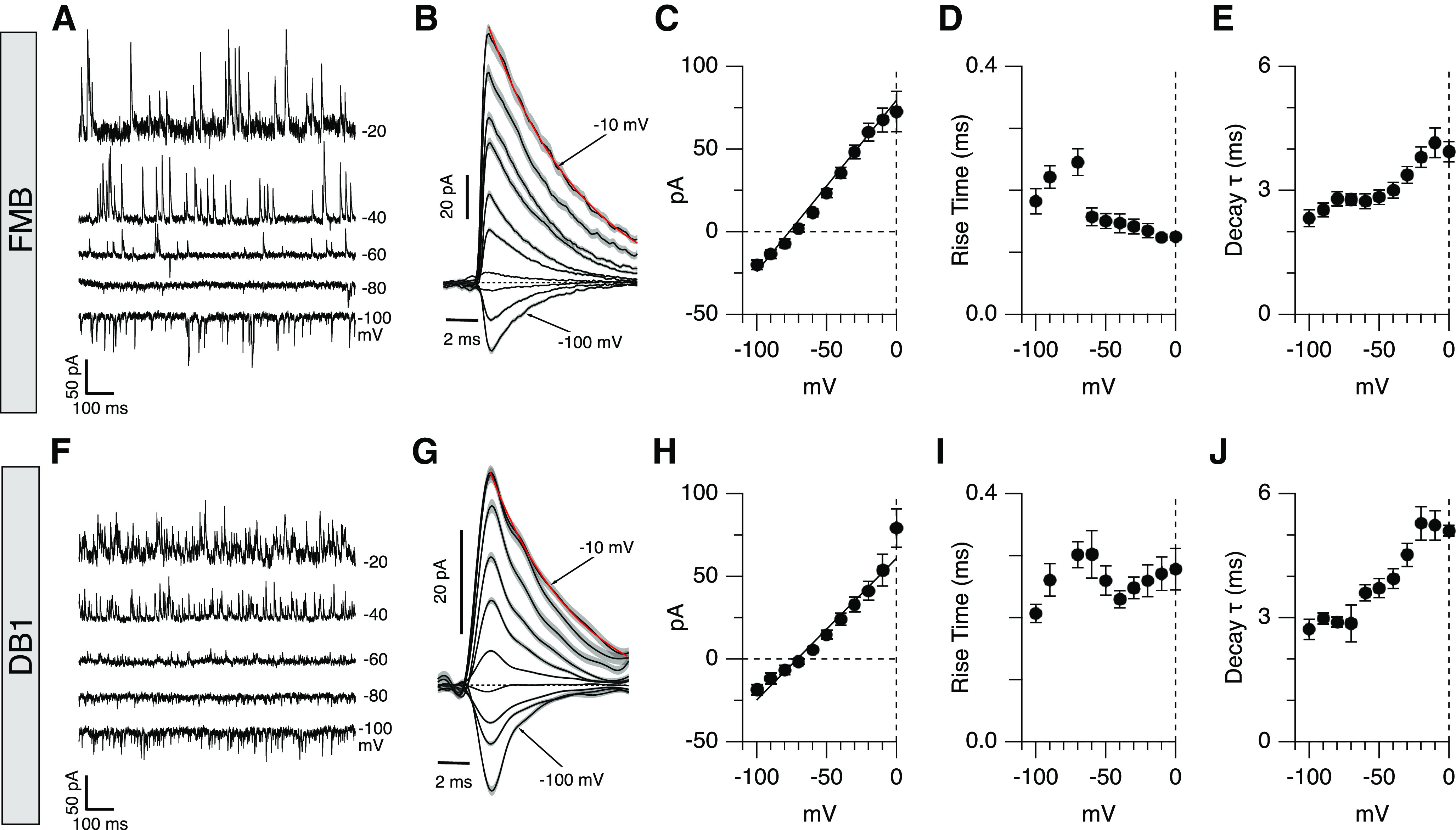
Properties of sIPSCs in FMB and DB1 bipolar cells. Quantification of sIPSC events in FMB (***A–E***) and DB1 (***F–J***) bipolar cells. ***A***, ***F***, Example traces showing sIPSCs in an FMB (***A***) and DB1 (***F***) cell at the holding potentials indicated to the right of traces. ***B***, ***G***, Average sIPSC events extracted from the same bipolar cells shown in ***A***, ***F*** using a matched template filter (average of 64–126 events per voltage for FMB, average of 70–130 events per voltage for DB1 cells). Gray shading shows SEM. Red traces show single exponential fits to event decay at −10 mV. ***C***, ***H***, I-V plots showing sIPSC amplitude as a function of voltage for a group of FMB (*n* = 20 cells; ***C***) and DB1 cells (*n* = 7 cells; ***H***). Note that events reverse close to the inhibitory reversal potential. ***D***, ***I***, Average sIPSC rise-time for the group of FMB (***D***) and DB1 (***I***) cells. ***E***, ***J***, Average sIPSC decay time constants for FMB (***E***) and DB1 (***J***) cells. All graphs show mean ± SEM.

To compare the properties of the sIPSCs in FMB and DB1 cells, we recorded sIPSCs at a range of holding potentials. Figure 4*A* shows sIPSCs in an FMB cell recorded at a range of holding potentials between −100 and −20 mV. At each potential, individual IPSCs were extracted using a matched template filter and averaged together to reveal the time course and amplitude of the IPSCs as a function of membrane potential (Fig. 4*B*; see Materials and Methods). This analysis was applied to a group of 20 FMB cells to produce the average I-V relations shown in Figure 4*C*. The mean sIPSC peak conductance for FMB cells was 0.97 nS and mean reversal potential was −76 mV, close to the calculated chloride reversal potential. The sIPSC rise-times were typical of synaptic events, with average 10–90% rise times ranging from 123 to 245 μs across the range of voltages tested (Fig. 4*D*). The decay phase of the sIPSCs was well fit by a single exponential function, with time constants exhibiting clear voltage dependence (τ = 2.1 ± 0.2 ms at −100 mV vs 4.0 ± 0.2 ms at 0 mV, *n* = 20, mean ± SEM *t* test, *p* < 0.0001; Fig. 4*B*,*E*). Similar analyses were conducted in seven DB1 cells revealing sIPSCs largely comparable to that of the FMB cells (Fig. 4*F–J*). For DB1 cells, the average conductance was 0.80 nS and average reversal potential was −73 mV (Fig. 4*H*). sIPSC rise times ranged from 206 to 278 μs and, like FMB cells, event decay time was voltage dependent [τ = 2.7 ± 0.2 ms at −100 mV vs 5.3 ± 0.3 ms at 0 mV (*n* = 7 cells), *t* test, *p* < 0.0001; Fig. 4*G*,*J*]. Taken together, the rapid kinetics and voltage dependence of the decay of the spontaneous glycinergic events in FMB and DB1 cells are consistent with the presence of GlyRα1 containing receptors, in accord with findings in Off-CBCs from other species (Gill et al., 2006; Ivanova et al., 2006). Additional evidence for selective expression of GLRA1 in DB1 and FMB cells is shown in Figure 8*A* below.

### Glycinergic input to Off-CBCs is driven by the On-pathway

**Figure 5.**
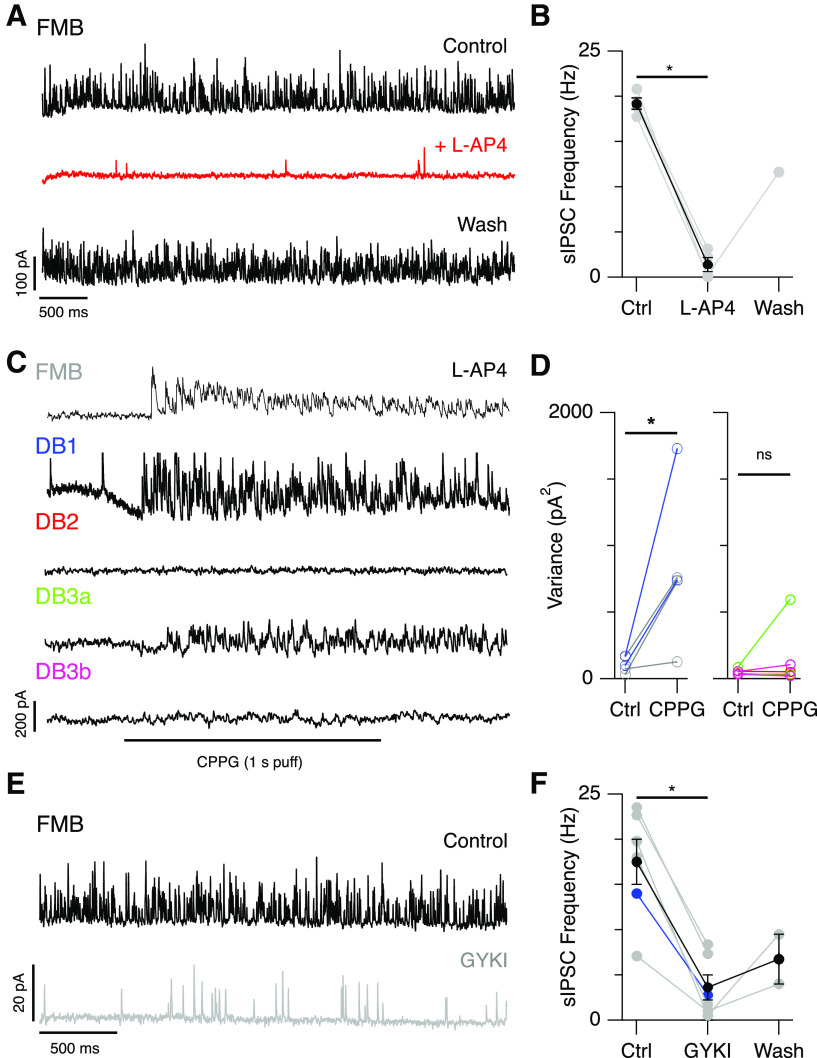
Inhibitory input to Off-CBCs is driven by the On-pathway. ***A***, Example traces showing sIPSCs in an FMB cell before, during, and after bath-application of L-AP4 (10 μm). ***B***, Effect of L-AP4 on sIPSC frequency for a group of FMB cells (Ctrl and L-AP4; *n* = 4 cells, washout; *n* = 1 cell, *p = 0.028, Wilcoxon test). Gray points represent individual cells, black points show mean ± SEM. ***C***, Example traces showing effect of puffing CPPG (600 μm) in the OPL in the presence of bath-applied L-AP4. CPPG puffs increase the frequency of sIPSCs in FMB, DB1, and DB3a cells but not in DB2 and DB3b cells. ***D***, Plots showing current variance before and after puff application of CPPG in the presence of L-AP4 for FMB/DB1 cells (left panel) and DB2/DB3a/DB3b cells (right panel). Data are from *n* = 3 FMB (gray), *n* = 2 DB1 (blue), *n* = 3 DB2 (red), *n* = 2 DB3a (green), and *n* = 2 DB3b (magenta); *p < 0.05 Wilcoxon rank test. Current traces in ***A***, ***C*** are at a step to 0 mV. ***E***, Example traces showing reduced frequency of sIPSCs in an FMB cell after bath application of the AMPA receptor antagonist, GYKI-53655 (10 μm). Holding potential is −60 mV. ***F***, Effect of GYKI on sIPSC frequency for a group of five FMB cells (gray points) and one DB1 cell (blue point). Partial washout was obtained in *n* = 2 FMB cells; *p = 0.016 with Wilcoxon rank test. Black points show mean ± SEM.

If glycinergic sIPSCs in Off-CBCs are driven by crossover inhibition from the On-pathway, their frequency should be modulated by activation or blockade of the mGluR6 receptors expressed by rod bipolar cells and On-CBCs. To test this prediction, we bath-applied L-AP4, an mGluR6 agonist that blocks tonic depolarization of rod and On-CBCs (Slaughter and Miller, 1983). Application of L-AP4 reduced the frequency of the sIPSCs in FMB cells by 93% [Ctrl 19.2 ± 1.3 vs L-AP4 1.4 ± 1.5 Hz (*n* = 4 cells), mean ± SD, *p* = 0.028, Wilcoxon test, partial washout in *n* = 1 cell; Fig. 5*A*,*B*]. Furthermore, puffing the mGluR6 receptor antagonist, CPPG, onto the OPL in the presence of L-AP4, to depolarize On-bipolar cells (Puthussery et al., 2009; Snellman et al., 2009), significantly increased sIPSC frequency in FMB and DB1 cells. Responses of DB3a cells to CPPG puffs were variable and difficult to assess because of their low frequency (Fig. 5*C*,*D*), while CPPG puffs failed to elicit IPSCs in DB2 and DB3b cells (Fig. 5*C*,*D*). Together, these results indicate that glycinergic sIPSCs in Off-CBCs arise through crossover inhibition driven by the On-pathway. These results further support the findings in Figure 2, suggesting that On-pathway mediated glycinergic inhibition is directed primarily toward FMB and DB1 cells.

Since mGluR6 receptors are present on the dendrites of rod bipolar and On-CBCs, the previous experiments do not determine which of these pathways drives glycinergic input to Off-CBCs. Anatomical studies indicate that AII-ACs synapse with both FMB (Grünert and Wässle, 1996; Grunert, 1997) and DB1 (Puthussery et al., 2011) cells. The CPPG puff experiments could depolarize AII-ACs either through gap-junctions with On-CBCs, or via the glutamatergic synapse from rod bipolar cells (Ghosh et al., 2001; Singer and Diamond, 2003). If the glycinergic inputs to Off-CBCs are driven exclusively by On-CBC to AII-AC coupling, these inputs should be insensitive to the AMPA receptor antagonist, GYKI-53655. Conversely, if the inhibitory input to Off-bipolar cells is driven by the rod bipolar cell to AII-AC pathway, then blocking glutamatergic transmission at the rod bipolar to AII-AC synapse (Fig. 1) with the AMPA receptor antagonist, GYKI-53655 (10 μm) should reduce IPSC frequency. We found that bath application of GYKI-53655 reduced the frequency of sIPSCs in FMB/DB1 cells by 81% (Ctrl 18±6.1 vs GYKI 3.6±1.4 Hz, *n* = 5 FMB cells and *n* = 1 DB1 cells, mean ± SD, *p* = 0.016, Wilcoxon rank test, partial washout in *n* = 2 FMB cells; Fig. 5*E*,*F*). Note that the sample size for DB1 cells was limited, given the low frequency with which these cells were encountered. These results suggest that the sIPSCs observed in FMB cells (and possibly DB1 cells) are likely to be driven, at least in part, by the rod bipolar cell → AII-AC pathway. However, these results cannot exclude the possibility that glycinergic input is also driven by other unknown On-pathway-driven glycinergic ACs. Note that at 10 μm concentration, GYKI-53655 suppresses AMPA receptor currents by >90% and kainate receptor currents by <5% (Wilding and Huettner, 1995). The incomplete suppression of the sIPSCs with GYKI seems could reflect glycinergic input driven by the On-CBC→AII-AC pathway, incomplete suppression by GYKI, or a minor Off-pathway-driven component.

### Voltage-gated currents distinguish macaque Off-CBC types

**Figure 6.**
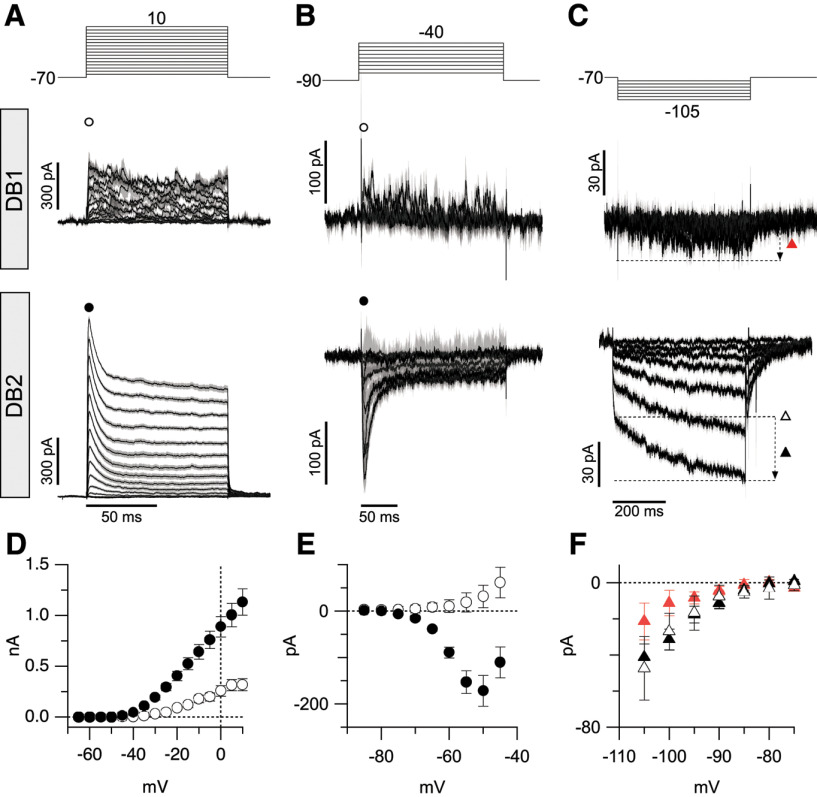
Voltage-gated currents in DB1 and DB2 bipolar cells. ***A–C***, Average leak-subtracted voltage-gated currents in DB1 and DB2 cells. Voltage step protocols are shown in top panels, and resulting currents from DB1 and DB2 cells are shown in the middle and bottom panels, respectively. Timing scale bar in lower panels applies to both cell types. Note prominent A-type potassium current, T-type calcium current, and K_IR_ and I_h_ currents in DB2 cells that is absent in DB1 cells. Data are normalized to max amplitudes. Gray shading in ***A–C*** shows ±1 SEM. ***D***, Comparison of peak outward potassium current in DB1 (open circles, *n* = 11) and DB2 (solid circles, *n* = 19) cells measured at a fixed time point 1.8 ms after step onset. Measurement time points are indicated with corresponding symbols in ***A***. ***E***, Comparison of inward current in DB1 (open circles, *n* = 4) and DB2 (solid circles, *n* = 13) cells at timepoints indicated by same symbols shown above traces in ***B*** (4 ms after step onset). ***F***, Comparison of average instantaneous (open triangle) and time-dependent inward current (black triangles) in DB2 cells (*n* = 13) and time-dependent current in DB1 cells (red triangles, *n* = 9). Instantaneous current was measured at a fixed time point (5.7 ms after step onset), time-dependent current is the difference between the minimum current amplitude and instantaneous current. Data are mean ± SEM.

The results presented thus far indicate that specific Off-CBC types receive higher levels of On-pathway-driven glycinergic input. Our next objective was to determine the intrinsic functional properties of the Off-CBC types that receive this input. In a prior study, we showed that the transient, diffuse bipolar cell types DB3a, DB3b and the sustained FMB cells, have distinct inventories of voltage-gated currents that shape their response properties (Puthussery et al., 2013). Here, we assessed the inventory of voltage-gated channels in DB1 and DB2 cells to determine whether their intrinsic properties favored transient or sustained signaling. First, we measured voltage-gated currents in DB1 and DB2 cells in response to depolarizing steps from −70 mV (Fig. 6*A*,*D*). DB2 cells displayed a large (>1 nA) A-type outward potassium current (I_KA_), which distinguished them from DB1 cells, which had smaller and relatively sustained I_K_ [peak amplitude at +10 mV (mean ± SD); DB1; 318 ± 196 pA, *n* = 11 cells vs DB2; 1132 ± 567 pA, *n* = 19 cells, Welch’s *t* test, *p* < 0.0001]. The large magnitude of I_KA_ also distinguishes DB2 cells from all other Off-CBC types described previously (Puthussery et al., 2013).

From a holding potential of –70 mV, there was little evidence for voltage-gated sodium or calcium currents during depolarizing steps in either DB1 or DB2 cells (Fig. 6*A*). However, both sodium channels and T-type calcium channels, which are expressed by bipolar cells (Puthussery et al., 2013), show significant steady-state inactivation at −70 mV. Therefore, we repeated the measurements from a holding potential of −90 mV, which is expected to relieve any such inactivation (Perez-Reyes, 2003; Puthussery et al., 2013; Fig. 6*B*,*E*). Under these conditions, DB2 cells exhibited a large, transient inward current that activated around −70 mV and reached an average peak amplitude of 171 ± 115 pA at ∼−50 mV (mean ± SD, *n* = 13 cells). A Boltzmann fit to the average I-V relation showed a V_1/2_ activation of −66 mV, rate (k) of 3.6 mV, and maximal conductance of 2.6 nS. The activation kinetics (10–90% rise time 1.9 ± 0.5 ms, measured at −50 mV, mean ± SD, *n* = 12 cells), time course of inactivation (single exponential fit, decay τ, 6.7 ± 1.9 ms, mean ± SD, *n* = 12 cells) and rate of recovery from steady-state inactivation (double exponential fit, fast phase, τ_1_ = 166 ± 33 ms, mean ± SD, *n* = 3 cells) were consistent with properties of T-type Ca_V_ channels, specifically the Ca_V_3.1 subunit (Perez-Reyes, 2003; Puthussery et al., 2013). Similar inward currents were absent from DB1 cells, indicating that they do not express voltage-gated sodium or T-type Ca_V_ channels.

We previously showed that inwardly-rectifying potassium (K_IR_) and hyperpolarization-activated currents are present in transient (DB3a and DB3b), but not sustained (FMB), macaque bipolar cells (Puthussery et al., 2013). We tested for the presence of these currents in DB1 and DB2 cells by applying hyperpolarizing voltage steps from −70 mV (Fig. 6*C*,*F*). DB1 cells displayed small slowly-activating inward currents during hyperpolarizing steps, with a maximum amplitude of −21±10 pA (*n* = 9; Fig. 6*C*,*F*). By contrast, DB2 cells displayed two nonlinear inward current components of approximately equal magnitude; an instantaneous component (−47 ± 17 pA, *n* = 13, solid symbols) and a time-dependent voltage-gated component (−41 ± 27 pA, *n* = 13, open symbols), consistent with the presence of K_IR_ and I_h_, respectively. Overall, these results support the idea that DB1 cells have relatively passive intrinsic properties, whereas DB2 cells exhibit active conductances, expected to make light responses more transient (bandpass).

### Identity and localization of voltage-gated ion channel subunits in DB1 and DB2 cells

**Figure 7.**
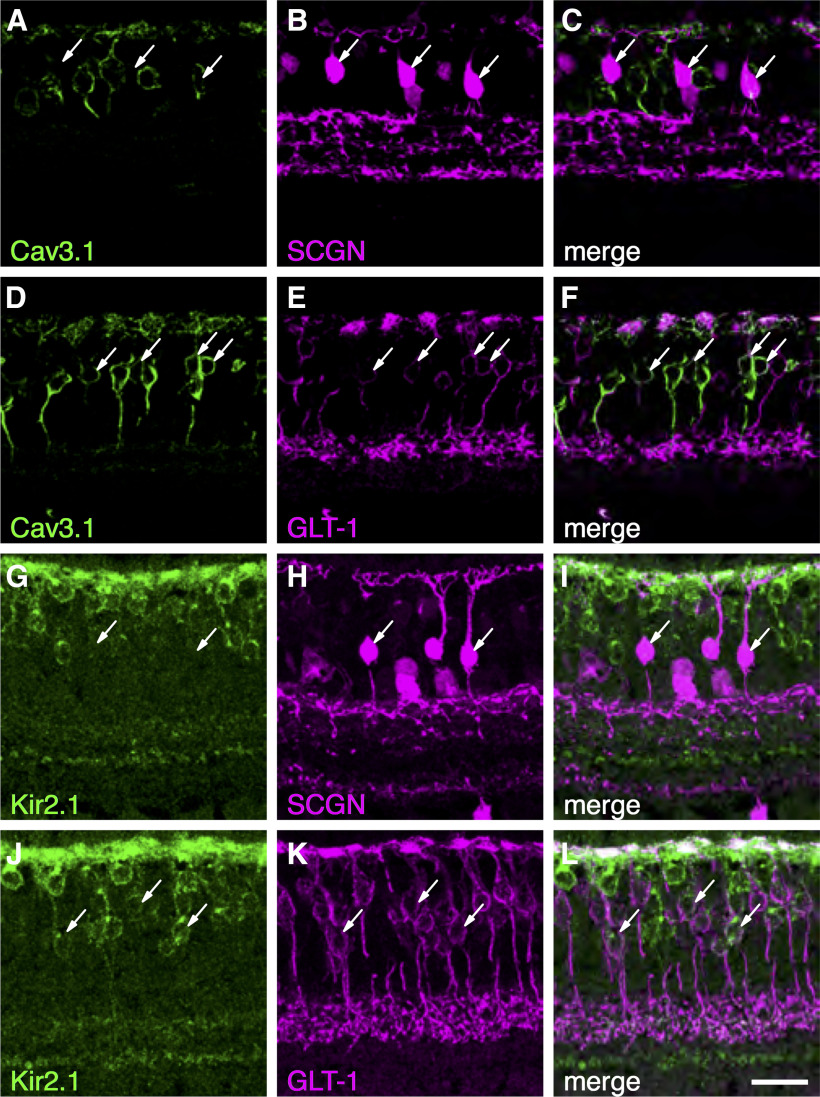
Voltage-gated ion channel subunits in primate DB1 and DB2 cells. ***A–F***, Localization of the T-type calcium channel subunit, Ca_V_3.1, in primate bipolar cells. Ca_V_3.1 is absent from SCGN+ DB1 cells (***A–C***, arrows). ***D–F***, A subset of GLT-1+ Off-bipolar cells express Ca_V_3.1, consistent with expression in DB2 cells (arrows). ***G–L***, The inward-rectifying potassium channel (K_IR_) subunit, Kir2.1, is absent from DB1 cells (***G–I***, arrows) but present in the soma, dendrites, and axon terminal boutons of GLT-1+ DB2 cells (***J–L***). Scale bar: 20 μm.

**Figure 8.**
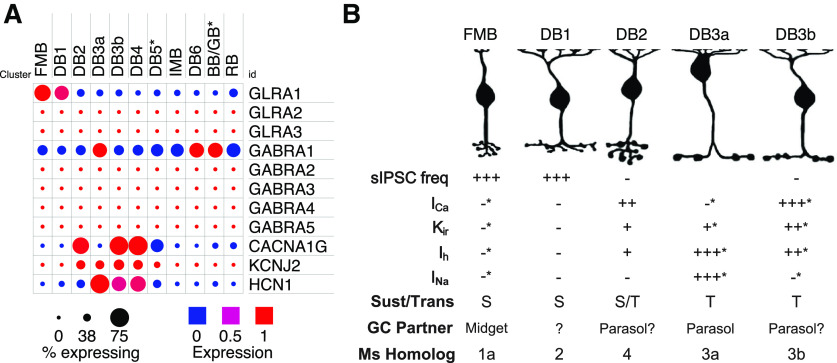
Summary of gene expression and physiological properties of macaque Off-bipolar cells. ***A***, Dot-plots showing comparative gene expression of glycine receptor (GLRA), GABA_A_ receptor (GABRA), and voltage-gated channel subunits in macaque peripheral bipolar cells. Dot color indicates expression level and dot diameter indicates the proportion of cells in the cluster in which the gene was detected. FMB and DB1 cells show higher levels of GLRA1 expression than other bipolar types. Raw data from Peng et al. (2019) plotted using the Broad Institute Single Cell Portal. ***B***, Summary of primate bipolar cell morphology, inhibitory inputs, physiological properties, voltage-activated currents, GC partners, and putative mouse (Ms) bipolar homologs. *Based on data from Puthussery et al. (2013). Homology based on expression data from Peng et al. (2019).

Our functional experiments indicated that DB2 cells exhibit I_h_, K_IR_, and T-type calcium currents, whereas these currents were absent in DB1 cells. To determine the molecular basis of these differences, we used immunohistochemistry to identify and localize the ion channel subunits underlying these currents (Fig. 7). In accord with our functional data, the Ca_V_3.1 subunit was localized to the somata of a subset of GLT-1-positive bipolar cells, but was absent from DB1 cells, which were identified with an antibody for secretagogin (SCGN; Puthussery et al., 2011; Fig. 7*A–F*). GLT-1 is present in both DB2 and FMB bipolar cells (Grünert et al., 1994); however, given the absence of T-type calcium currents in FMB (Puthussery et al., 2013) cells, it is likely that the DB2 cells express the Ca_V_3.1 subunit. We also sought to determine the origin of the inward currents in DB2 cells (Fig. 7*G–L*). We found the inward-rectifier potassium channel subunit, Kir2.1, in DB2 cells, but not in DB1, cells. This channel subunit was abundant in the dendrites and present to a lesser extent in the soma, axon and axon terminal boutons. These molecular differences are further supported by transcriptomic data showing higher levels of expression of CACNA1G (Cav3.1) and KCNJ2 (Kir2.1) in DB2 compared with DB1 cells (Fig. 8*A*).

The voltage-gated currents and immunohistochemical data shown here, and in our previous study (Puthussery et al., 2013), allow us to distinguish five primate Off-CBC types based on the amplitude and kinetics of I_K_, as well as the presence or absence of I_Na_, T-type I_Ca_, I_h_, and K_IR_ currents. Of these five types, DB1 and FMB were most similar with respect to their inventory of voltage-gated channels, but they can be readily distinguished by their characteristic dendritic and axonal morphologies. Our results suggest that bipolar cells with axon terminals in stratum 1 of the IPL (FMB and DB1 cells) have relatively passive membrane conductances and thus are expected to exhibit relatively sustained physiological responses. Conversely, bipolar cells with axon terminals in stratum 2 of the IPL (DB2, DB3a, and DB3b) have a variety of active conductances (T-type I_Ca_, I_Na_, and I_h_) that likely contribute to their transient physiological response properties (Puthussery et al., 2013). Taken together, our results suggest that On-pathway-driven glycinergic inhibition arises at least in part from the rod-pathway and is biased toward sustained Off-CBC types. The functional properties of the glycinergic IPSCs are consistent with expression of the α1 GlyR subunit. Single-cell RNA sequencing data provide additional support for higher expression of α1 GlyR transcript (GLRA1) in DB1 and FMB cells compared with other Off-CBC types (Fig. 8*A*).

## Discussion

The AII-AC represents a critical hub in the rod and cone pathways. Evidence from other mammals suggests that AII-AC output synapses are strongly biased toward specific Off-CBC types (McGuire et al., 1984; Tsukamoto and Omi, 2017; Graydon et al., 2018). Here, we have shown that a subset of primate Off-CBC types (DB1 and FMB cells) receive higher levels of On-pathway-driven glycinergic inhibition, that is likely driven at least in part by the rod bipolar cell→ AII AC synapse. The kinetics of the glycinergic events are consistent with involvement of the α1 GlyR subunit, a result supported by high levels of GLRA1 transcript in DB1 and FMB cells. The lack of active conductances, and axon terminal stratification suggest that DB1 cells, like FMB cells, have sustained temporal responses. Our results therefore predict that in primate retina, sustained type Off-CBCs may have higher scotopic sensitivity, and greater susceptibility to crossover inhibition under photopic light levels, than transient Off-CBC types.

### On-to-Off crossover glycinergic inhibition to specific Off-CBC types

We found that specific Off-CBC types receive high levels of spontaneous inhibitory input that was abolished by a GlyR antagonist, and strongly suppressed by blocking On-bipolar signaling. This crossover glycinergic inhibition was strongly suppressed by GYKI and thus, when taken together with anatomic evidence for AII-AC input to DB1 and FMB cells (Wässle et al., 1995; Grünert and Wässle, 1996; Grunert, 1997; Puthussery et al., 2011), suggests involvement of the primary rod pathway (rod→ rod bipolar cell→ AII-AC→Off-CBC; Fig. 1). The sustained Off-CBC types, FMB and DB1, had the highest levels of glycinergic input, whereas the transient types, DB2 and DB3b, received the least (summarized in Fig. 8*B*). In mouse retina, AII-AC synapses are preferentially directed toward type 2 Off-CBCs (CBC2; 50–69%; Tsukamoto and Omi, 2013; Graydon et al., 2018). Like FMB and DB1 cells, the putative mouse homologs, the type 1a and 2 CBCs (Fig. 8*B*; Peng et al., 2019) stratify in S1 of the IPL, and have relatively sustained physiology (Baden et al., 2013; Borghuis et al., 2013; Franke et al., 2017). In cat retina, the S1-stratifying CBa1 Off-CBCs cells are the major recipient of AII-AC input (McGuire et al., 1984). Overall, our results in macaque, taken together with studies from other mammals, suggest a conserved motif, where AII-AC outputs are primarily targeted toward sustained Off-CBC pathways. Routing through the low-pass sustained pathways may be important to ensure the faithful transmission of transient and sustained output signals from AII-ACs (Balakrishnan et al., 2015; Graydon et al., 2018).

A further emerging pattern is that each Off-CBC type primarily receives either primary or tertiary rod pathway input (Demb and Singer, 2012). For example, the mouse bipolar types, 3a, 3b, and 4 directly contact rods (Mataruga et al., 2007; Haverkamp et al., 2008) but receive little AII-AC input. Our results align with these findings, in that DB3b cells, which showed the lowest levels of glycinergic input, are the only Off-CBC type that synapse directly with rods (Tsukamoto and Omi, 2014). Notably, in macaque, direct rod-to-Off-CBC input occurs less frequently than in mouse, a result that may explain the lower contribution of the tertiary rod pathway to scotopic signaling in primates (Grimes et al., 2018a).

### Glycinergic input to Off-CBCs is mediated by α1 GlyRs

GlyRs are comprised of one of four α subunits (α1, α2, α3, or α4) together with a modulatory β subunit (Lynch, 2009). The decay kinetics of α1 containing receptors are faster than those containing α2 or α3 (Singer et al., 1998). The rapid activation (∼200–300 μs) and decay kinetics (2–5 ms) of the glycinergic sIPSCs we recorded in Off-CBCs are consistent with the fast kinetics of α1β receptors and similar to glycinergic IPSCs in Off-CBCs of other mammals (Ivanova et al., 2006; Schubert et al., 2008; Wässle et al., 2009; Eggers and Lukasiewicz, 2011; Graydon et al., 2018). The GlyRα1 subunit is concentrated in the Off sublamina of the IPL, where it has been localized to contacts between AII-ACs and Off-CBCs (Sassoe-Pognetto et al., 1994; Grünert and Wässle, 1996). In macaque, GlyRα1 puncta are concentrated in S1 of the IPL and the majority (∼75%) are associated with AII-AC synapses (Grünert and Wässle, 1996). GlyRα1 puncta are localized at sites where DB1 and FMB cells contact AII-AC lobular appendages, and such puncta are present to a lesser extent at DB3a axon terminals (Jusuf et al., 2005; Puthussery et al., 2011). The higher transcript levels of GLRA1 (GlyRα1) in FMB and DB1 cells (Fig. 8*A*), further suggests biased output of AII-ACs toward these cells, especially since no other GlyR subunits are detected at high levels in any Off-CBCs (Fig. 8*A*; raw data from Peng et al., 2019). Taken together, our data provide functional evidence for GlyRα1 expression on FMB and DB1 cells. Based on the anatomic evidence cited above, AII-ACs are a likely presynaptic partner, although inputs from other glycinergic ACs cannot be excluded.

### Voltage-gated channels in sustained versus transient Off-CBCs

We previously showed that CBCs that drive Off-midget and Off-parasol GCs differ in their inventory of ion channels (Puthussery et al., 2013). Bipolar cells that provide input to parasol GCs had prominent Na_V_ or T-type Ca_V_ currents, K_IR_ and prominent I_h_ currents, which contribute to their transient temporal response properties. Conversely, FMB cells, which provide excitatory input to midget GCs, were relatively devoid of these channels. The results here extend on our previous analyses. We found that DB2 cells, which elaborate their axon terminals in S2 of the IPL showed prominent T-type Ca_V_ currents and corresponding expression of Ca_V_3.1, whereas the S1-stratifying DB1 cells (Puthussery et al., 2011) lack these channels. Similarly, K_IR_ currents and the corresponding channel subunit, Kir2.1, were evident in DB2, but not DB1 cells. These data further support the idea that specific voltage-dependent ion channels contribute to the response properties of sustained versus transient bipolar cell types. T-type Ca_V_, I_h_, and K_IR_ channels are associated with CBCs that stratify near the middle of the IPL (Puthussery et al., 2013), findings further corroborated by data from single-cell sequencing (Fig. 8*A*; Peng et al., 2019). Overall, our results indicate that signals transmitted through the primary rod pathway are directed toward Off-CBCs that are tuned to lower temporal frequencies. Primate rod signals are slowest under low scotopic conditions and speed up as background illumination increases (Grimes et al., 2018a). Thus, selectively routing rod signals into bipolar cells with low-pass temporal tuning would serve to preserve the fidelity of slow rod signals near scotopic threshold.

### Implications for scotopic signaling in the magnocellular and parvocellular pathway

Do all primate GCs participate in scotopic vision? Both Off-midget (parvocellular) and Off-parasol (magnocellular) GCs receive scotopic input (Lee et al., 1997; Field et al., 2009; Ala-Laurila and Rieke, 2014; Grimes et al., 2018a). Indeed, scotopic input to Off parasol GCs was strongly suppressed by blockade of the On-pathway (Grimes et al., 2018a; their Fig. 6*E*), indicating these inputs arise from the primary rod pathway. DB3a cells likely provide the major excitatory input to Off-parasol cells (Jacoby and Marshak, 2000) . Although DB3a cells receive some On-pathway-driven input (Fig. 4*C*,*D*), because of the sparsity of these cells we could not unequivocally determine the pharmacological basis of this inhibition. One possibility is that On-pathway-driven inhibition in these cells is mediated by GABA receptors. Indeed, DB3a cells express high levels of GABRA1 (GABA_A1_), but lower levels of GLRA1 (GlyRα1) than DB1 and FMB cells (Fig. 8*B*). Although anatomic studies suggest that DB3a cells may receive some direct input from AII-ACs (Jusuf et al., 2005; Grünert and Wässle, 2006), it is possible that rod signals are routed directly from AII-ACs to Off-parasol GCs (Bordt et al., 2006). In mouse retina, rod signals may be routed directly from AII-ACs to Off GCs at scotopic threshold (Arman and Sampath, 2012) whereas Off-CBCs are engaged at higher scotopic levels (Mazade and Eggers, 2013). Primate Off-parasol GCs express GlyRα1 (Lin et al., 2000; Peng et al., 2019) and thus it is possible that they too receive scotopic input directly from AII-ACs. Indeed, Off parasol cells receive On-pathway-driven glycinergic input (Crook et al., 2014). Further studies are needed to clarify the circuit that transmits rod input to primate Off-parasol GCs.

We found that DB2 and DB3b cells do not receive glycinergic input or express significant levels of GLRA1 raising the possibility that the Off-GCs downstream of these cells receive less scotopic input. In mice, scotopic sensitivity varies across Off-GC types, with thresholds of some Off-GCs unaffected by blockade of the primary rod pathway (Völgyi et al., 2004). Further electron microscopy reconstructions and circuit analyses are required to quantify AII-AC output to different primate Off-CBCs and Off-GCs, and to establish the postsynaptic partners of the various Off-CBCs. Functional recordings from different types of primate Off-bipolar and Off-GC would also clarify whether specific primate output pathways are scotopically blind or exhibit lower scotopic sensitivity.

## Synthesis

Reviewing Editor: Kirill Martemyanov, The Scripps Research Institute Decisions are customarily a result of the Reviewing Editor and the peer reviewers coming together and discussing their recommendations until a consensus is reached. When revisions are invited, a fact-based synthesis statement explaining their decision and outlining what is needed to prepare a revision will be listed below. The following reviewer(s) agreed to reveal their identity: Alapakkam Sampath. Note: If this manuscript was transferred from JNeurosci and a decision was made to accept the manuscript without peer review, a brief statement to this effect will instead be what is listed below.

N/A
